# Efficacy of high dose Vitamin D supplementation in improving serum 25(OH)D among migrant and non migrant population: a retrospective study

**DOI:** 10.1186/s12913-016-1798-3

**Published:** 2016-10-13

**Authors:** Usha Gowda, Thilanga Ruwanpathirana, David P. S. Fong, Ambika Kaur, Andre M. N. Renzaho

**Affiliations:** 1 Global Health and Society Unit, School of Public Health and Preventive Medicine, Monash University, Victoria, Australia; 2 Centre for Cardiovascular Research and Education in Therapeutics, Department of Epidemiology and Preventive Medicine, School of Public Health and Preventive Medicine, Monash University, Victoria, Australia; 3 Doutta Galla Community Health Service, Kensington, VIC Australia; 4 Centre for International Health, Burnet Institute, Victoria, Australia; 5 School of Social Sciences and Psychology, University of Western Sydney, Locked bag 1797, Penrith, 2751 NSW Australia

**Keywords:** Vitamin D deficiency, Serum 25(OH)D, High dose cholecalciferol

## Abstract

**Background:**

Higher dose of vitamin D supplementation 50000 IU is required for those whose serum 25(OH)D levels are 50 nmol/L and below. The increment in serum 25(OH)D though not significantly affected by race, sex or age it is negatively correlated to the baseline 25(OH)D concentration. This study investigated whether the mean increase in serum 25(OH)D will be higher among participants with lower baseline 25(OH)D levels and whether the duration of supplementation has an influence on the serum 25(OH)D achieved.

**Methods:**

A clinical audit of patients’ medical records from a community health centre in Melbourne for the period 01.01.2010 to 31–12.2012 was undertaken. Paired sample t test was used to determine difference in pre and post dose serum 25(OH)D. Simple and multiple linear regressions were used to examine the association between the difference in pre and post dose serum 25(OH)D and duration of supplementation and baseline serum 25(OH)D, adjusting for socio-demographic factors.

**Results:**

A total of 205 patients were included in the study. Mean difference in serum 25(OH)D was highest 52.8 nmol/L (95 % CI: 46.63–58.92) among those whose serum 25(OH)D was below 25 nmol/L at baseline. Baseline 25(OH)D alone accounted for 13.7 % of variance in the effect size (F_(2, 202)_ = 16.0. *p* < 0.001), with the effect size significantly higher among participants with a baseline 25(OH)D level of 25–49 nmol/L (β = 11.93, 95 % CI: 0.48, 23.40, *p* < 0.05). Mean serum 25(OH)D difference was highest, 47.53 nmol/L (95 % CI: 40.95–54.11) when measured within 3 months of supplementation. Duration of supplementation explained 2.9 % of the variance in the effect size (F _(1, 203)_ = 6.11, *p* < 0.05) and there was an inverse relationship between the length of supplementation and mean pre and post supplementation serum 25(OH)D difference (β = −1.45, 95 % CI: −2.62, −0.29, *p* = 0.014).

**Conclusion:**

Following 50000 IU vitamin D3 for 12 months mean serum 25(OH)D increase was highest among those whose baseline serum 25(OH)D was lower. Migrants especially dark-skinned are at a high risk for vitamin D deficiency in Australia. High dose vitamin D3 50000 IU (cholecalciferol) is effective in achieving sufficient serum 25(OH)D among these populations who tend to have lower baseline serum 25(OH)D.

## Background

Vitamin D deficiency (VDD) is a significant public health issue worldwide. VDD is commonly defined as serum 25(OH)D below 50 nmol/L [1]. While there have been different cut-off points proposed to define VDD, the widely accepted is that of Endocrine society, International osteoporosis and US department for health which proposed that serum 25(OH)D levels should be greater than 75 nmol/L particularly in older adults [2–5]. Low levels of vitamin D have been found to be associated with deleterious health including increased risk for fractures, functional limitations and chronic diseases [2]. More recently low vitamin D has been linked to mental health conditions including schizophrenia, altered immunity and other autoimmune diseases [6].

However VDD is unequally distributed and those with dark skinned population in high Northern 37° N or low Southern 37° S latitudes seem to be affected more than light skinned population [7, 8]. VDD is common in immigrant groups from developing countries living in western countries, including Northern Europe, USA and Australia [9]. Possible risk factors for the high levels of VDD include dark skin and decreased sun exposure after settling in the host country [10].

Although sun is the main source of vitamin D, production of vitamin D is influenced by the season, latitude, [7] skin colour and skin exposure [11]. Vitamin D3 is found naturally in small quantities such as oily fish, eggs and fortified foods such as margarine and some low-fat milk products [12]. Therefore ongoing supplements may be necessary for people at high risk of vitamin D deficiency [13].

While vitamin D supplementation is needed to achieve a desired threshold of serum 25(OH)D concentrations among deficient individuals, there is no universally accepted threshold at which initiating vitamin D supplementation would achieve the greatest impact. In Victoria, Australia for those whose serum levels are 50 nmol/L and below recommendations are to supplement with higher dose 50000 IU per month and serum 25(OH)D to be rechecked after 3 months and then at 12 months [6] as it takes 3–5 months for serum 25(OH)D to plateau [14, 15].

Although the incremental increase in serum 25(OH)D is not significantly affected by race, sex or age it is negatively correlated to the baseline 25(OH)D concentration [14, 16]. In a study by Trang et al. subjects taking 4000 IU vitamin D2 or vitamin D3, the effect of vitamins D2 and D3 at increasing serum 25(OH)D concentrations diminished progressively at above 50 nmol/L basal 25(OH)D [17]. There is little evidence for the effect of high dose vitamin D supplementation on the increase in serum 25(OH)D at lower baseline serum 25(OH)D levels. Therefore the aim of this study was to examine whether the effect of 50,000 IU vitamin D3 once a month over 12 months would be greater among subjects with lower baseline 25(OH)D (<50 nmol/L) than those with higher baseline 25(OH)D (>50 nmol/L) and whether this was dependent on the length of treatment. We hypothesised that 1) post vitamin D supplementation change in serum 25(OH)D levels will be greater among participants with lower baseline 25(OH)D levels and 2) increase in serum 25(OH)D levels will be inversely proportional to the length of vitamin D supplementation.

## Method

### Design and setting

The study was a retrospective audit of medical records. The study design has been described elsewhere [18–20]. Briefly an audit of patient records at a major metropolitan community health centre in the western region of Melbourne was undertaken. The centre provides primary, community and specialist mental health services to the people across the City of Melbourne and Moonee Valley City Council. Of these 48 % from the City of Melbourne and 23 % from Moonee Valley City were born overseas [21]. The centre offers health assessment for immigrants arriving as family migrants, asylum seekers and refugees with the vast majority arriving from Asia, Africa and Middle Eastern countries.

### Inclusion criteria and Vitamin D supplementation protocol

Our initial contact with the health practitioners was in 2013 and we chose to include all adults aged 18 years and above who attended the health centre between 01.01.2010 and 31.12.2012. The intent of 3 years time frame was to include a representative sample of the migrant population who attended the community centre for the initial health assessment and follow up. Inactive patients such as deceased and those patients who had not recorded their country of birth during registration were excluded from the study.

Only those who were prescribed a high dose 50,000 IU Vitamin D3, had serum 25(OH)D measured both pre and post vitamin D supplementation was included in the study. Individuals who had serum 25(OH)D below 50 nmol/L were prescribed a high dose 50000 IU vitamin D3. However few individuals whose serum 25(OH)D was above 50 nmol/L were also supplemented with 50000 IU vitamin D3. Patients were prescribed one capsule of 50000 IU vitamin D3 for 12 months. However, those individuals whose serum 25(OH)D was below 25 nmol/L one 50000 IU vitamin D3 capsule per week for 4 weeks followed by one capsule per month was prescribed. Of the 2,187 patients, 1,217 had serum vitamin D measured in the time period (01.01.2010–31.12.2012) covered by the audit. A total of 205 patients met our inclusion criteria and was included in the study.

### Primary outcome and study variables

The primary outcome was the post serum 25(OH)D levels following vitamin D supplementation and mean serum 25(OH)D difference in pre and post vitamin D supplementation.

### Independent variables

#### Baseline 25(OH)D

For each patient, serum 25(OH)D and the date of test recorded in the electronic database was obtained. Names of the laboratories which undertook patients’ vitamin D testing was obtained from the patients’ database. Information on the biochemical assay used in the analysis of serum 25(OH)D was obtained from the referring laboratories. Vitamin D testing was predominantly undertaken by a single pathology which used Diasorin immunoassay for measuring serum 25(OH)D of the patients.

For the purpose of this study following classification was used: vitamin D sufficiency > 75 nmol/L; sub-optimal levels 50–75 nmol/L; vitamin D insufficiency 25–50 nmol/L, moderate vitamin D deficiency 15–25 nmol/L, and severe vitamin D deficiency < 15 nmol/L [22]. Due to small sample size in cells we combined the sufficiency and suboptimal groups as suboptimal-to-sufficient levels (50–75 nmol/L) and severe deficiency and moderate deficient groups were categorised as moderate-to-severe deficient group (<25 nmol/L).

#### Duration of treatment

The number of days between the date of supplementation and the first post dose serum 25(OH)D test within 12 months period was considered as the duration of vitamin D supplementation. Since our audit was for a three year period the date of vitamin D supplementation varied across the population.

#### Socio-demographic variables

In our recent study we found that vitamin D deficiency post-migration was closely linked with the region of origin [18]. Therefore it was important to control for migration status. We obtained country of birth for each patient that was recorded at the time of patients’ registration at the health centre. The patients were classified as Migrant (those born overseas) and Non migrant (those born in Australia). Similarly, various studies have found that the prevalence of vitamin D deficiency increases significantly with age and are greater in women [23]. The effect of age and gender was controlled for in our analysis. Date of birth and gender for each patient was obtained from the medical record. The patients were classified into following age groups; 18-34 years, 35-54 years and those above 55 years [24].

## Data analysis

Data was analysed using STATA 12 (Stata, Texas). To account for the seasonal variation in serum 25(OH)D adjusted serum 25(OH)D was computed using the formula: [25]

$$ \mathit{\mathsf{Adjusted}}\ \mathit{\mathsf{v}\mathsf{itamin}}\ \mathit{\mathsf{D}}=\left( Log\ \left(\mathit{\mathsf{v}}\mathit{\mathsf{i}}\mathit{\mathsf{t}}\ \mathit{\mathsf{D}}\right)+\left[\mathit{\mathsf{overall}}\ \mathit{\mathsf{mean}}\ \mathit{\mathsf{log}}\left(\mathit{\mathsf{v}}\mathit{\mathsf{i}}\mathit{\mathsf{t}}\ \mathit{\mathsf{D}}\right) - \mathit{\mathsf{mean}}\left(\mathit{\mathsf{log}}\ \mathit{\mathsf{v}}\mathit{\mathsf{i}}\mathit{\mathsf{t}}\ \mathit{\mathsf{D}}\right)\kern0.15em \mathit{\mathsf{sampled}}\ \mathit{\mathsf{i}\mathsf{n}}\ \mathit{\mathsf{same}}\ \mathit{\mathsf{month}}\right]\right) $$

Paired sample t test was used to determine the difference in pre and post dose serum 25(OH)D. Simple and multiple linear regressions were used to examine the association between the difference in pre and post dose serum 25(OH)D and duration of supplementation and baseline serum 25(OH)D, adjusting for socio-demographic factors.

## Results

The study included 205 participants of whom only 11.7 % had suboptimal level-to-sufficient (50–75 nmol/L) serum 25(OH)D, 45.85 % had insufficient (25–49 nmol/L) serum 25(OH)D and 42.44 % were classified as moderate-to-severe (<25 nmol/L) vitamin D deficient (Table 1). The average age of the study population was 43.89 ± 15.36 years and the average duration between the date of supplementation and the date of first post dose vitamin D testing was 5.73 ± 3.22 months.

**Table 1:** Mean serum 25(OH)D difference for pre and post vitamin D supplementation across all demographic characteristics

Variables	Number (Percent)	Serum 25OHD Pre-dose Mean (SD)	Serum 25OHD Post-dose Mean (SD)	Mean serum difference 95 % Class Interval	*P* value
All	205 (100)	30.39 (14.43)	72.27 (26.06)	41.88 (38.14–45.63)	<0.05
Serum 25 OHD Category					
50–75 nmol/L	24(11.70)	58.22 (8.66)	82.57(22.48)	24.34(16.54–32.15)	<0.05
25–49 nmol/L	94(45.85)	35.08 (6.68)	71.37(23.35)	36.28 (31.48–41.08)	<0.05
Below 25 nmol/L	87(42.43)	17.63 (4.56)	70.41 (20.19)	52.77 (46.63–58.92)	<0.05
Duration of Supplementation					
3 months and below	64(31.21)	28.21(13.04)	75.74(24.02)	47.53(40.95–54.11)	<0.05
4–6 months	66(32.19)	32.13(14.54)	74.70(24.94)	42.57(35.58–49.96)	<0.05
7–9 months	42(20.48)	28.92(15.08)	68.89(25.51)	39.90(31.83–47.97)	<0.05
10-12 months	33(16.09)	32.92(15.13)	65.00(31.47)	32.08(22.81–41.35)	<0.05
Age Category					
18–34	70(34.14)	28.64(12.87)	70.26(28.46)	41.79(35.51–48.08)	<0.05
35–54	79(38.53)	28.67(14.08)	70.58(24.72)	41.80(35.86–47.75)	<0.05
55 and above	56(27.31)	35.20(15.83)	77.32(24.89)	42.11(34.19–50.03)	<0.05
Gender					
Female	137(66.82)	30.35(15.38)	71.03(26.61)	40.68(36.05–45.31)	<0.05
Male	68 (33.17)	30.47(12.39)	74.78(24.92)	44.31(37.82–50.80)	<0.05
Migrant status					
Non migrant	56 (27.31)	34.34(14.26)	72.81(26.68)	38.46(30.68–46.24)	<0.05
Migrant	149(72.68)	28.90(14.26)	72.02(25.91)	43.17(38.88–47.45)	<0.05

At baseline, more than three quarters (>85 %) of participants had serum levels below 50 nmol/L. Following vitamin D supplementation, the post supplementation serum 25(OH)D averaged 82.7 ± 22.48 nmol/L, 71.37 ± 23.35 nmol/L and 70.4 ± 20.19 nmol/L among participants whose serum 25(OH)D at baseline was 50–75 nmol/L, 25-49 nmol/L and below 25 nmol/L respectively. At 3 months post vitamin D supplementation, serum 25(OH)D was 75.74 ± 24.02 nmol/L and at 4–6 months serum 25(OH)D was 74.70 ± 24.90 nmol/L. Rise in serum 25(OH)D levels was lower at 7–9 months measuring 68.89 ± 25.51 nmol/L and 65.00 ± 31.47 nmol/L when measured at 10–12 months (Table 1).

### Effect size

The overall effect size (mean difference of 25(OH)D between post and pre supplementation) of the vitamin D supplementation of 50,000 IU was 41.9 nmol/L (95 % CI: 38.1, 45.6). Post treatment, the mean difference in serum 25(OH)D was highest 52.8 nmol/L (95 % CI: 46.63–58.92) among those whose serum 25(OH)D was below 25 nmol/L at baseline followed by those between 25–49 nmol/L which was 36.3 nmol/L (95 % CI:31.48–41.08) and 24 nmol/L (95 % CI:16.54–32.15) among those who had serum 25(OH)D between 50–75 nmol/L at base line (Table 1). Baseline 25(OH)D alone accounted for 13.7 % of variance in the effect size (F_(2, 202)_ = 16.0. *p* < 0.001), with the effect size significantly higher among participants with a baseline 25(OH)D level of 25–49 nmol/L (β = 11.93, 95 % CI: 0.48, 23.40, *p* < 0.05) and below 25 nmol/L (β = 28.43, 95 % CI: 16.88, 39.98, *P* < 0.001) than those with 50–75 nmol/L (Table 2). These results remained consistent after controlling for duration of treatment, gender, age, and migration status (Table 2). Mean serum 25(OH)D difference was highest, 47.53 nmol/L (95 % CI: 40.95–54.11) when measured within 3 months of supplementation, 42.57 nmol/L (95 % CI:35.58–49.96) at 4–6 months, 39.90 nmol/L (95 % CI:31.83–47.97) at 7–9 months and lowest when measured at 10–12 months 32.08 nmol/L (95 % CI:22.81–41.35) (Table 1). Duration of supplementation explained 2.9 % of the variance in the effect size (F _(1, 203)_ = 6.11, *p* < 0.05). That is, there was an inverse relationship between the length of supplementation and mean pre and post supplementation serum 25(OH)D difference (β = −1.45, 95 % CI: −2.62, −0.29, p = 0.014). These results remained consistent after controlling for baseline 25(OH)D level, gender, age, and migration status (Table 2). The effect of age, gender, and migration status was negligible and non-significant.

**Table 2:** Multiple linear regression for the predictors of mean serum 25OHD difference following vitamin D supplementation

Variables	Unadjusted		Adjusted	
	β	95 % CI	β	95 % CI
Serum 25(OH)D Category				
50-75 nmol/L	Ref			
25-49 nmol/L	11.83*	0.48–23.39	12.73*	1.16–4.30
Below 25 nmol/L	28.43***	16.88–39.98	28.40***	16.73–40.08
Duration of Supplementation				
(Months)	−1.45*	−2.62–-0.29	−1.18*	−2.29–-0.06
Age (years)	−0.016	−0.26–0.22	0.09	−0.15–0.33
Gender				
Female	Ref			
Male	3.62	−4.33–11.59	4.52	−3.02–12.07
Migrant status				
Non migrant	Ref			
Migrant	4.70	−3.69–13.11	1.87	−6.60–10.35

*p < 0.05; ***p < 0.001

## Discussion

To the best of our knowledge this is the first retrospective study in Australia to examine the effect of high dose, 50000 IU oral vitamin D3 (cholecalciferol) supplementation on mean serum 25(OH)D among migrant and non migrant population. Our hypothesis that there would be an inverse relationship between pre-supplementation serum 25(OH)D and the pre-and post vitamin D supplementation mean serum difference in 25(OH)D was confirmed.

Studies evaluating effect of high dose vitamin D on serum 25(OH)D are limited. However, our findings are similar to those reported by Talwar et al. [26] and Garrett-Mayer and colleagues [27]. For example, Garrett-Mayer and colleagues [27] undertook a study among African and white men who were treated with 4000 IU/day for 1 year. Participants in both the groups had serum 25(OH)D below 50 nmol/L at baseline. Participants with the lowest baseline serum 25(OH)D had the largest increase by 2 months [27]. Similar inverse association for baseline serum 25(OH)D and increase in serum 25(OH)D was seen among young and old men following vitamin D supplementation 800 IU/day for 8 weeks [16]. According to Heany et al. [28] the concentration of serum 25(OH)D in response to vitamin D3 is biphasic, that is, a rapid increase occurs at low vitamin D3 concentrations and a slower response occurs at higher concentrations.

We also hypothesised that the post-vitamin D supplementation 25(OH)D levels will be inversely associated with the length of supplementation. This hypothesis was confirmed, but the effect was significantly smaller than that associated with baseline 25(OH)D levels. This finding is inconsistent with the literature. In an observational study by Shab-Bidar et al. [29] the effects of increasing doses (400 IU, 800 IU, 1700 IU and 3500 IU/day) of vitamin D3 at lower baseline serum 25(OH)D values on the serum 25(OH)D was determined. They reported that the increase in mean serum 25(OH)D was higher, 24.3 ± 34.0 nmol/L after 11 months of supplementation compared to mean serum 25(OH)D, 17 ± 32 nmol/L after 4 months of supplementation. However unlike the study by Shab-Bidar et al. [29] where the same group of patients were followed at 4 months and 11 months period, we reported mean serum difference for those who were tested within 3 months, 4–6 months, 7–9 months and 10–12 months of vitamin D supplementation. Shab-Bidar et al. undertook meta-regression analysis based on randomized clinical trials (RCTs) in adults to review the influence of supplementation-related factors (dose and duration) and patient-related factors (age and baseline 25[OH]D) on changes in serum levels of 25(OH)D [30]. An immediate increase in pooled mean difference of serum 25(OH)D was found in patients with baseline <50 nmol/L reaching a plateau after 6 months indicating substantial increase in serum 25(OH)D within few months of vitamin D supplementation.

Possible factors influencing the mean serum difference at different time intervals in our study could be the noncompliance of the patients with regards to vitamin D supplementation. Due to retrospective nature of this study we did not have information on the patients’ compliance with the vitamin D supplementation. Moreover individuals in our study whose serum 25(OH)D was below 25 nmol/L at baseline were prescribed one 50000 IU vitamin D3 per week for a month followed by one per month. This disparity in dosing regimen might have resulted in a higher serum 25(OH)D achieved within 3 months and also by those whose serum 25(OH)D was below 25 nmol/L at baseline. In a randomized dose response study among UK male athletes, 17 out of 30 (57 %) had mean serum levels 51 ± 24 nmol/L at baseline, of which 20 % (6 out of 30) were classified as deficient or severely deficient. Supplementation with 40000 IU vitamin D3 per week for 6 weeks resulted in higher serum 25(OH)D, 98 ± 14 nmol/L compared to those who were supplemented with 20000 IU vitamin D3 per week, 79 ± 14 nmol/L [31]. However there was no significant difference in serum levels after 12 weeks of supplementation with both groups achieving serum 25(OH)D above 75 nmol/L i.e. 91 ± 24 nmol/l (40000 IU) and 85 ± 10 nmol/L (20000 IU). This study further reported that there was no additional advantage of 40000 IU/week for a target serum of 75 nmol/L. Whether this implies to our study is unclear due to the diverse population which included migrants, non migrants, different age groups, men and women unlike the previous study [30] which included only active male athletes.

Patient disease state such as gastrointestinal abnormalities, kidney and liver disease also affects the metabolism of vitamin D [32]. It has been emphasized that the genetic make-up of subjects may play an important role in that subjects with different vitamin D binding protein (DBP) genotypes have different responses to the same vitamin D dose [33]. Although only those individuals in our study whose serum 25(OH)D was 50–75 nmol/L at baseline achieved serum 25(OH)D above 75 nmol/L,individuals whose serum 25(OH)D was below 50 nmol/L achieved an average serum25(OH)D levels of 70 nmol/L. Thus 50000 IU vitamin D3 supplementation for 12 months was effective in achieving vitamin D sufficiency i.e. serum 25(OH)D above 50 nmol/L among VDD adult population.

Findings from our study showed that supplementation with high dose i.e. 50000 IU of vitamin D3 cholecalciferol restored serum levels to sufficient levels i.e. above 50 nmol/L among migrants and non migrants especially for those who have lower baseline serum 25(OH)D. Migrants especially those who are dark skinned have a higher prevalence of vitamin D deficiency (<50 nmol/L) in Australia [34–36]. Therefore high-dose oral cholecalciferol may be more practical and cost-effective than low dose regimen in treating VDD. The cost of high dose 50000 IU vitamin D3 is $20 for 10 tablets while the cost of the low-dose supplements is about $30 for 300 capsules at retail pharmacies in Australia [37].

Our study found that the pre and post vitamin D supplementation mean difference in serum 25(OH)D did not vary by migration status, age, and gender. A study in Britain among adults aged 65–85 both males and females achieved similar serum 25(OH)D 75.6 and 72 nmol/L following vitamin D supplementation of 100,000 IU/4 months for 5 years [38]. Aloia et al. in their study among 18–65 years found no significant sex differences on serum 25(OH)D in response to vitamin D among black and whites males and females [14]. In contrast, various studies have reported the influence of age. For example in Harris et al. [16] the magnitude of change in serum 25(OH)D after 8 weeks of 800 IU Vitamin D supplementation was almost similar, 22.5 nmol/L and 22.1 nmol/L in the young and the old men respectively. In a cross sectional study of Chinese men, mean serum 25(OH) D levels was highest among participants aged from 40 to 59, while it was lowest in the 20–39 age groups [39]. The precise vitamin D dose taken by the participants were unknown, they were stated as taking vitamin D >250 IU/day. Whether differences in the sun exposure behaviour among the old and young men (working indoors) accounted for variations in serum 25(OH)D levels was not clear.

### Limitations

Due to the retrospective nature of the study, our findings may have been influenced by potential confounding factors. We did not have the data for Body Mass Index (BMI) on all patients hence this variable was not included in our study. Obesity has been associated with decreased bioavailability of dietary vitamin D [32]. Lack of information on outdoor activities, clothing style whether patients fully covered their skin with clothing or not when they were in the sun, use of sunscreen all of these could either enhance or reduce serum 25(OH)D levels depending on their sun exposure behaviours. We were unable to determine whether race had an effect on increments in 25(OH)D levels due to lack of information on physical characteristics such as skin colour. Determinants of race such as skin colour are hard to measure in general practice due to limited resources and time required to undertake such tests [40]. Therefore we were unable to undertake subgroup analysis to examine the association between the race and variations in serum 25(OH)D levels following vitamin D supplementation. We were unable to verify patients’ compliance to vitamin D which may have led to variations in serum 25(OH)D levels.

## Conclusions

Vitamin D deficiency is a crucial health disparity among dark sinned migrants in Australia. Vitamin D synthesis through the skin is limited in these migrants and the dietary sources of vitamin D are also limited. High dose 50000 IU vitamin D3, cholecalciferol remains a practical replacement for low dose regimen in terms of efficacy and cost effectiveness. Supplementation of high dose 50000 IU vitamin D3 to treat severe vitamin D deficiency is mandated in Victoria. It is timely that other states in Australia follow this example to treat severe vitamin D deficiency among migrants as well as Australian population. However currently there are insufficient studies that have examined the efficacy of high dose 50000 IU vitamin D supplementation in treating VDD. Future randomised controlled trials that examine the efficacy and safety of high dose vitamin D supplementation in attaining sufficient serum 25(OH)D are required.

## Abbreviations

BMI: Body mass index
DBP: Vitamin D binding protein
RCT: Randomised clinical trial
VDD: Vitamin D deficiency
